# Restricted kinematic total knee arthroplasty provided better functional outcomes and higher satisfaction rates for Asians of genu varum with apex distal joint line over unrestricted kinematic total knee arthroplasty

**DOI:** 10.1186/s42836-025-00318-x

**Published:** 2025-07-02

**Authors:** Jia Yi Loh, Ming Han Lincoln Liow, Glen Purnomo, Merrill Lee, Jerry Yongqiang Chen, Hee-Nee Pang, Keng Jin Darren Tay, Seng-Jin Yeo

**Affiliations:** 1 Department of Orthopaedic Surgery, Singapore General Hospital, Singapore, 169608 Singapore; 2 St Vincentius a Paulo Catholic Hospital, Surabaya, 60241 Indonesia; 3 National Hospital, Surabaya, 60227 Indonesia

**Keywords:** Coronal plane alignment of the knee, CPAK, Knee, Arthroplasty

## Abstract

**Background:**

Coronal Plane Alignment of the Knee (CPAK) phenotypes I, II, and IV can achieve favorable soft tissue balance following kinematic total knee arthroplasty (KA-TKA). Given that this classification was developed from a Caucasian population, limited studies have evaluated clinical outcomes following restricted vs unrestricted KA-TKA in South-East Asian patients, this study aimed to: (1) outline the prevalence of CPAK types in a South-East Asian population, (2) compare clinical outcomes of patients undergoing restricted versus unrestricted KA-TKA.

**Methods:**

Prospectively collected data from 232 patients who underwent KA-TKA between 2015 and 2018 were reviewed. The prevalence of CPAK in our cohort was determined using preoperative medial proximal tibial (MPTA) and lateral distal femoral (LDFA) angles measured from long-limb radiographs. Unrestricted calipered KA-TKA targeted equal bone cuts while restricted KA-TKA utilized intraoperative navigation to restrict tibia varus to 4°. Patients were assessed preoperatively, at 6 months, and 2 years using the Knee Society Score, Oxford Knee Score, and Short-Form 36. Postoperative satisfaction and expectation fulfillment were recorded. Intra- and interclass correlation of all radiographic measurements and both parametric and non-parametric statistical analysis were used.

**Results:**

The prevalence of CPAK in our cohort: I (47.8%), II (30.6%), III (9.1%), IV (7.8%), V (3.9%), VI (0.9%), VII (0%), VIII (0%) and IX (0%). Intra- and interclass correlation of radiographic measurements were excellent at 0.98 (95%CI: 0.95–0.99, *P* < 0.01). Subgroup analysis of CPAK I patients demonstrated that restricted KA-TKA had better KSS objective (*P* = 0.04), a higher proportion of satisfied patients (*P* = 0.02) at 6 months, and better OKS (*P* = 0.03) than unrestricted KA-TKA.

**Conclusion:**

CPAK I was the most prevalent phenotype in an Asian population. CPAK I patients undergoing restricted KA-TKA had better functional outcomes and satisfaction rates than those who underwent unrestricted KA-TKA. Future studies should focus on evaluating outcomes of different alignment strategies to personalize treatment for Asian CPAK phenotypes.

## Introduction

There is no consensus for total knee arthroplasty (TKA) coronal alignment targets due to robotic augmentation of human abilities and differing surgeon preferences [1]. Mechanical alignment (MA) aims to recreate a neutral Hip-Knee-Angle (HKA) within 1.5° of 180° by achieving orthogonal cuts of femur and tibia and subsequently balancing soft tissue to achieve rectangular gaps in flexion and extension. Kinematically aligned total knee arthroplasty (KA-TKA) aims to restore the native knee joint kinematics through “resurfacing” and respects the natural variability in knee anatomy across individuals without compromising implant survivorship [1–4]. The concept of restricted KA-TKA targeted postoperative HKA within 3° of the neutral axis to avoid excessive varus component positioning, which could affect implant survivorship [1]. Currently, both restricted and unrestricted KA-TKA showed comparable outcomes to MA-TKA [3, 4]. However, emerging studies in Caucasian and Asian patients have shown that KA-TKA achieved superior outcomes for certain phenotypes of knees based on the coronal plane alignment of the knee (CPAK) compared to MA-TKA [5–7]. With the plethora of emerging alignment strategies and an enhanced understanding of patients’ pre-arthritic anatomy, Macdessi et al. introduced a novel CPAK classification to address the coronal deformity variability across the population using the HKA and Joint Line Obliquity (JLO) [8]. However, the CPAK classification has yet to be extensively studied in the Asian population, and to date, has not been widely studied in the Southeast Asian population [7, 9, 10]. To offer “personalised arthroplasty”, surgeons will need to understand the underlying demographics and phenotypes of the loco-regional patients to offer patients alignment strategies that improve functional outcomes, reduce pain without compromising implant longevity. This study aimed to delineate differences in functional outcomes between restricted and unrestricted KA-TKA within a South-east Asian population of predominantly CPAK I knee.

## Materials and methods

This study was exempted from our local institutional review board review. Prospectively collected data were obtained from 232 patients who underwent KA-TKA between 2015 and 2018 by a single fellowship-trained arthroplasty surgeon in a single institution. All patients underwent TKA using P.F.C Sigma implants (DePuy Orthopaedics Inc., Warsaw, IN, USA). Demographic data, including age, gender, side of surgery, comorbidities, and body mass index, were recorded. A power analysis was conducted using a two-tailed *t*-test for independent means. An effect size of 0.82 was derived using the minimal clinically important difference (MCID) of the Oxford Knee Score (OKS) and standard deviation as determined by Clement et al. [11]. To achieve an 90% power, with a significance level of 0.05 and an effect size of 0.82, a total sample size of 27 per arm was required to detect significant differences between the two independent means, with an allocation ratio of 1:1. Liow et al. [12] performed a separate power analysis in 2014 which showed a minimum sample size of 29 knees to detect significant differences between knee arthroplasty groups within a South-East Asian demographic.

### Radiological measurements

**Fig. 1:**
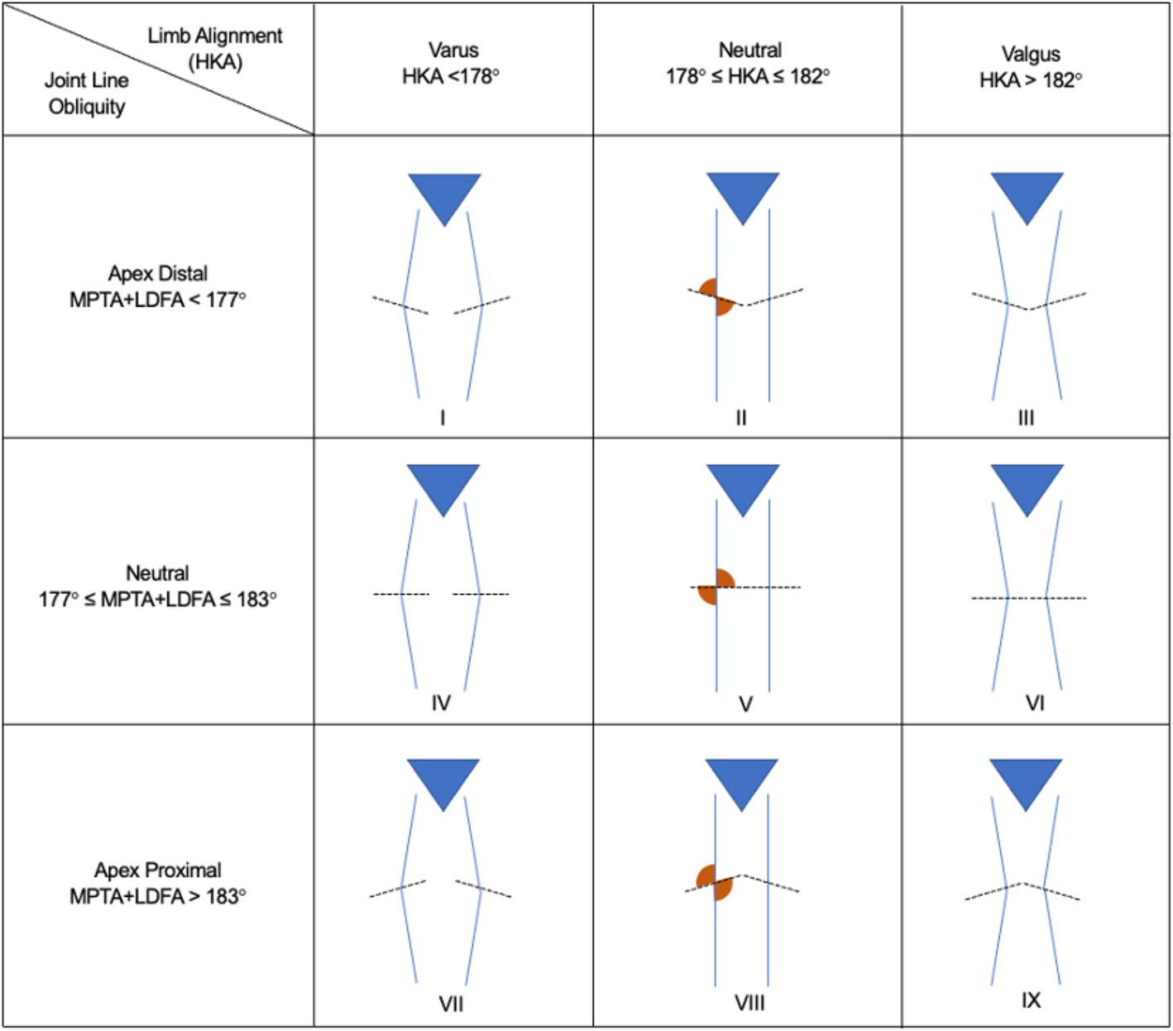
Coronal plane alignment of the knee (CPAK) classification according to limb alignment and joint line obliquity

All patients underwent preoperative long-limb radiographs. Patients were then classified into CPAK types based on HKA and JLO as measured on the long-limb radiographs. Medial proximal tibial (MPTA) and lateral distal femoral (LDFA) angles measured on long limb radiographs were used to determine the HKA and JLO [10, 13]. The LDFA was defined as the lateral angle formed by the femoral mechanical axis and the distal femoral joint line. The MPTA was defined as the medial angle formed by the tibial mechanical axis and the proximal tibial joint line. The HKA was determined by subtracting the LDFA from the MPTA and dividing the radiographs into neutral, varus, and valgus alignment. The JLO was determined by the sum of the MPTA and LDFA, and divided the radiographs into apex distal, neutral, and apex proximal. Subsequently, radiographs were categorised into CPAK classifications I-IX as proposed initially by MacDessi et al., with CPAK 1 being a varus HKA with a JLO with apex distal (Fig. 1) [8]. All measurements were performed by three authors, and the inter- and intraclass correlation of radiographic measurements was excellent at 0.98 (95% CI: 0.95–0.99, *P* < 0.01).

### Restricted vs Unrestricted KA-TKA

Unrestricted KA-TKA was performed by cutting equal femur and tibia cuts according to Howell’s bone caliper technique [14] to restore the native pre-arthritic alignment. Equal amounts of distal femur bone resection were performed after removal of cartilage with a curette. The native posterior condylar axis was visualized, considering posterior condyle wear, and the PCA was used to perform the proximal tibia cut. The 4-in-1 cut was completed, and bone fragments were measured using a caliper to ensure that equal amounts of bone had been resected. For restricted KA-TKA, the tibia cut was kept within 4° and was guided by intraoperative navigation to achieve balanced gaps in both flexion and extension. Postoperatively, all patients underwent protocolised rehabilitation programs based on institutional guidelines. Postoperative long-limb radiographs were obtained, and postoperative HKA angle was measured by the intersection of a line drawn from the center of the femoral head to the femoral intercondylar notch and another line from the tibial interspinous point to the tibial mid-plafond [3].

### Patient-reported outcomes

Preoperative patient-reported outcome measures (PROMs), including functional and objective knee society scores (KSS), OKS, and short-form 36 (SF-36) physical component score (PCS), were collected prior to KA-TKA. Patients were then followed up 6 months and 2 years postoperatively. In each visit, satisfaction rates, expectation fulfilment rates, and PROMS, including functional and objective KSS, OKS, and SF-36 PCS, were recorded.

### Comparison of outcomes

Independent samples *t*-test and chi-square test were used to compare restricted and unrestricted KA-TKA in the most prevalent CPAK I type. A *P*-value < 0.05 was deemed significant. All statistical calculations were performed using IBM SPSS software, version 25 (Armonk, NY, USA).

## Results

### Demographics and CPAK classification

Preoperative phenotypes were predominantly CPAK I (47.8%). The remaining minority of CPAK phenotypes were II (30.6%), III (9.1%), IV (7.8%), V (3.9%), and VI (0.9%). No CPAK VII, VIII, or IX were found in our population. Within the CPAK I phenotype (*n* = 111), 81 patients underwent restricted KA-TKA and 30 patients underwent unrestricted KA-TKA. Mean postoperative HKA angle was 0.95° ± 2.31° for restricted KA-TKA and 2.42° ± 2.37° for unrestricted KA-TKA.

Within the CPAK I phenotype, there were no significant differences in terms of BMI, Charlson comorbidity index, gender, and side of surgery between patients who underwent restricted KA-TKA compared to unrestricted KA-TKA. There were also no significant differences in functional outcomes preoperatively between the two groups. However, the mean age of patients who underwent restricted KA-TKA was 67.7 years, 3.1 years less than those who underwent unrestricted KA-TKA (Table 1).

**Table 1:** Comparison of postoperative outcomes for patients with CPAK I phenotype following restricted vs unrestricted kinematic total knee arthroplasty

Kinematic total knee arthroplasty	Restricted	Unrestricted	*P*-value
	***n* = 81**	***n* = 30**	
**Preoperative**			
Age: mean (years)	67.7	70.8	0.034
Body mass index; mean (kg/m^2^)	27.1	27.4	0.78
Charlson comorbidity index; mean	0.3	0.4	0.515
Operated side; Right (%)	55.6	63.3	0.461
Sex; Females (%)	74.1	76.7	0.78
Knee Society Score, functional	53.6	48.7	0.166
Knee Society Score, objective	34	30.8	0.29
Oxford Knee Score	24.7	24.1	0.752
SF36 Physical Component Score	34.4	31.7	0.175
**6 months postoperatively**			
Knee Society Score, functional	77.6	74.3	0.372
Knee Society Score, objective	88.5	83.6	0.044
Oxford Knee Score	41.8	40.6	0.26
SF36 Physical Component Score	50.2	47.2	0.091
Met expectations (%)	96	88.9	0.178
Satisfied (%)	98.7	88.9	0.024
**2 years postoperatively**			
Knee Society Score, functional	82.9	78	0.271
Knee Society Score, objective	90.3	89.7	0.796
Oxford Knee Score	43.5	41.2	0.026
SF36 Physical Component Score	51.4	50.8	0.732
Met expectations (%)	95.6	100	0.55
Satisfied (%)	97.1	100	1

### Functional outcomes in restricted vs unrestricted KA-TKA within CPAK I

Patients who underwent restricted KA-TKA had better overall functional scores compared to unrestricted KA-TKA and had significantly better objective KSS scores and satisfaction rates at 6 months compared to patients who underwent unrestricted KA-TKA (*P* < 0.05). However, the significantly better objective KSS scores did not hit the MCID [15]. Patients within CPAK 1 who underwent restricted KA-TKA initially showed similar OKS at 6 months, but subsequently showed improved OKS at 2 years compared to unrestricted KA-TKA, but this similarly did not hit the MCID threshold [16]. Subsequently, the improved objective KSS scores and satisfaction rates plateau at 2 years, showing no difference between the two groups.

## Discussion

The main finding in this study was that within the CPAK I phenotype, restricted KA-TKA provided superior short and mid-term benefits in terms of functional outcomes and satisfaction rates compared to unrestricted KA-TKA. To date, robotic-assisted arthroplasty and navigation have not led to an improvement in clinical scores and satisfaction rates [17, 18]. To address this problem, Howell et al. proposed the kinematically aligned knee, restoring native joint kinematics and improving functional scores compared to addressing all arthritic knees equally by mechanical alignment [2]. However, some studies showed that component loosening was a potential concern for patients with significant varus knees in going for kinematical alignment [19, 20]. Vendittoli et al. thus developed the concept of restricted kinematic alignment, preventing component loosening in cases of severe varus deformity [21]. Still, the ideal alignment for knee arthroplasty is a continuously moving target, made even more complex with the concept of different native alignment phenotypes as described by the CPAK classification. The adoption of robotic-assisted TKA has allowed surgeons to accurately achieve a desired alignment, yet there has been no consensus on an ideal alignment strategy for any given patient. Functional alignment (FA) is gaining traction as an alternative alignment technique, prioritising soft tissue balancing by aiming to restore the plane and obliquity of the native joint line. Recently, Clark and Jeffrey et al. both showed significant functional outcome improvements using FA-TKA as compared to both MA-TKA and KA-TKA [22, 23]. Similarly, our results show that a greater focus on soft tissue balancing after restricting the cuts could have prevented laxity associated with an unrestricted KA-TKA, allowing for similar functional outcome improvements as seen in FA-TKA, particularly for CPAK I patients. Further studies need to be done to evaluate the ideal alignment strategy that maximises both patient satisfaction and functional outcomes for different CPAK groups.

This study also shows that the original CPAK phenotyping may be valuable in determining patient-specific arthroplasty targets, and this may be the key to achieving superior patient outcomes with robotic navigation in knee arthroplasty. Agarwal et al. initially reported that a change in the patient’s native joint line and CPAK classification using mechanically aligned RA-TKA does not significantly affect satisfaction rates 1 year postoperatively [24]. However, Pangaud and Clarke et al. both recently showed that restoration of CPAK in patients with preoperative genu varus is associated with improved functional outcome scores at 2-year follow-up, however, the improvement was less evident for patients with preoperative neutral alignment [25, 26]. Further understanding of the CPAK classification and its repercussions on alignment strategies will allow surgeons to tailor the coronal alignment targets to maximise functional outcome measures, paving the way for personalised arthroplasty.

This study is one of the first to evaluate knee phenotypes in a Southeast Asian population based on the CPAK classification. CPAK I phenotype was the most common phenotype present in the population of arthritic knees, with varus constitutional alignment and an apex-distal JLO. The differences in CPAK distribution within an Asian population and the resultant ideal alignment target have yet to be extensively studied. The initial CPAK classification as reported by MacDessi et al. for the Australian population was predominantly Type II with neutral HKA and neutral JLO. However, this study showed a predominant Type I CPAK phenotype distribution. Several Asian populations in Japan and India have shown similar CPAK distribution with a predominant Type I phenotype [7, 9, 27]. This difference in phenotype distribution may yield key information in terms of surgical planning and alignment targets. This study showed that within a South-east Asian population of predominant CPAK Type I knees, restricted KA-TKA showed short and mid-term functional outcomes superiority and improved satisfaction rates compared to unrestricted KA-TKA. Given that CPAK classification only analyses the knee in a single coronal plane, future studies should consider sagittal alignment parameters like tibial slope or axial plane parameters such as the femur component rotation, which have been shown to affect functional and radiological outcomes after TKR [28]. Other areas of research include monitoring long-term functional outcomes and survivorship, as well as the influence of coronal deformity severity. Moreover, further studies on the distribution of the CPAK category are necessary to understand the diverse CPAK classes within certain ethnic groups within a population and their impact on functional outcomes. Azmi et al. recently showed that the Malay population in Singapore was less likely to be CPAK-1, possibly accounting for differences in functional outcomes compared to the Chinese and Indians within the same population [29]. Azmi et al. also identified that the same alignment strategy may not be ideal for bilateral TKA, given that only 45% of patients within the Singapore population had the same CPAK category bilaterally [29]. Further studies are to be aimed at deriving personalised arthroplasty through understanding the native anatomy in all planes and deriving the most ideal alignment strategy for each patient individually.

There are some limitations to this study. First, the retrospective design without a control group was used. This restricts the generalizability of the results. Second, the sample sizes of unrestricted KA-TKA within the CPAK I distribution were significantly smaller than restricted KA-TKA. This is due to surgeon preference during the period studied and the evolution of the alignment strategy of the surgeon. Further studies with larger sample sizes will be required for more precise subgroup analysis. Thirdly, complication rates were not recorded and analysed for this study. Although revision rates due to loosening are a concern in unrestricted KA-TKA [17, 30]. Future studies should include a complications analysis to further delineate the surgical targets for the respective CPAK phenotypes. Lastly, the CPAK phenotyping in this study did not include patients who did not undergo TKA, and this could under-represent the TKA population. However, it would be representative of the multi-racial, ethnically diverse Singaporean population with osteoarthritis, warranting Orthopaedic review.

## Conclusion

The CPAK classification has allowed surgeons to determine preoperative parameters that will delineate future surgical planning [8, 14]. Restricted KA-TKA showed improved short-term functional outcomes and satisfaction rates within the CPAK I phenotype of arthritic knees compared to unrestricted KA-TKA. As robotic navigation continues to permeate arthroplasty practices worldwide, there is a greater need to define and outline arthroplasty targets. The concept of personalized arthroplasty using robotic navigation techniques that allow retaining of the patient’s native knee kinematics in combination with patient-specific implants will allow surgeons to determine the appropriate surgical targets preoperatively. Thus, further studies should continue to identify specific patient phenotypes that will benefit from different surgical targets, to achieve personalized arthroplasty to improve patient outcomes without compromising implant longevity.

## Data Availability

The datasets generated and/or analyzed during the current study are not publicly available due to patient confidentiality and protection under the Personal Data Protection Act in Singapore.
